# A Ferroptosis-Related LncRNA Signature Associated with Prognosis, Tumor Immune Environment, and Genome Instability in Hepatocellular Carcinoma

**DOI:** 10.1155/2022/6284540

**Published:** 2022-08-18

**Authors:** Jie Lian, Chaoyu Zhang, Haibo Lu

**Affiliations:** ^1^Department of Outpatient Chemotherapy, Harbin Medical University Cancer Hospital, Harbin, 150000 Heilongjiang Province, China; ^2^Department of Gynecology and Obstetrics, Medical Faculty, Justus-Liebig-University Giessen, 35390, Giessen, Germany

## Abstract

**Background:**

Ferroptosis is an iron-dependent form of cell death. In this study, we identified ferroptosis-related long noncoding RNAs (FRlncRNAs) to investigate their association with hepatocellular carcinoma (HCC) in prognosis, tumor immune environment, and genome instability.

**Methods:**

Transcriptome profile data of HCC were retrieved from a public database. FRlncRNAs were identified by co-expression analysis. Patients were randomly divided into training and test cohorts. Univariate Cox analysis and Least Absolute Shrinkage and Selection Operator (LASSO) Cox regression were performed to construct a risk model. Patients were divided into high- and low-risk groups based on the risk model. AUC and C index were used to assess the risk model. Survival analysis, immune status, and genome instability were compared between the two groups.

**Results:**

Sixteen FRlncRNAs were identified and used to construct an FRlncRNA signature for the risk model. The Kaplan-Meier analysis revealed that patients in the high-risk group had poorer overall survival than patients in the low-risk group. The area under curve of the risk model was 0.879, 0.809, and 0.757 in the training cohort and 0.635, 0.688, and 0.739 in the test cohort at 1, 2, and 3 years, respectively. The risk model was an independent prognostic predictor and showed excellent prediction of prognosis compared with clinicopathological features. For the differentially expressed ferroptosis-related genes, many enriched metabolic pathways were identified in the functional enrichment analysis. Immune cells such as CD8+ T cells, macrophages M1, natural killer cells, and B cells, which may be associated with antitumor immune responses, differed between the high- and low-risk groups. Genome instability based on the risk model was also explored. A total of 61 genes were differently mutated between the two risk groups, and among them, *TP53*, *HECW2*, *TRIM66*, *MCTP2*, and *KIAA1551* had the most significant mutation frequency differences.

**Conclusion:**

The FRlncRNA signature is closely related with overall survival, tumor immune environment, and genome instability in HCC.

## 1. Introduction

The Global Cancer Statistics in 2020 (GLOBOCAN 2020) ranked liver cancer as the sixth cancer among newly diagnosed cancers and the third leading cause of cancer death, with an incidence of 906,000 cases and 830,000 deaths annually [1]. Hepatocellular carcinoma (HCC) is the most common liver cancer, accounting for 90% of primary liver cancer cases, caused mainly by hepatitis B, hepatitis C, alcohol, and toxic exposure [2, 3]. Because most patients are diagnosed when the cancer is at an advanced stage, surgical removal can be performed on only 5%–15% of patients who have early-stage cancers, and chemotherapy and immunotherapy are better options for the other patients [4]. However, the problem of drug resistance of chemotherapy is extremely challenging. The emergence of immunotherapy provided a new approach to cancer treatment, but immunotherapy is not effective in a significant number of patients. Therefore, predicting prognostic biomarkers and targeting patients for whom immunotherapy is most likely to be effective is important for precision treatment.

In 2012, Dixon et al. [5] were the first to recognize ferroptosis as an iron-dependent form of cell death induced by the small molecules erastin and RSL3. Compared with other cell death patterns, such as apoptosis, necrosis, and autophagy, ferroptosis has specific morphological, biochemical, and genetic characteristics. Three essential features of ferroptosis are the loss of lipid peroxide repair capacity by the phospholipid hydroperoxidase GPX4, the availability of redox active iron, and oxidation of polyunsaturated fatty acid-containing phospholipids [6]. Many studies have shown that ferroptosis plays an important role in cancer, including ovarian, lung, and liver cancers [7–9]. Because of the increased iron demand of cancer cells during growth, tumor cells are more sensitive than normal cells to ferroptosis [10]. The tumor suppressor p53 gene, which is mutated in approximately 50% of cancers, hypoxia-inducible factors, and mesenchymal-like states, is also involved in ferroptosis regulation [11]. Although a number of underlying mechanisms have been uncovered, many challenges remain, including finding the effector molecule of ferroptosis, uncovering the interaction between ferroptotic and non-ferroptotic regulated cell death, and assessing patient suitability for proferroptotic therapy [12].

Long noncoding RNAs (lncRNAs) are a class of non-protein coding transcripts that are >200-nucleotides long. They account for almost 80% of the human transcriptome. Recent studies have shown that lncRNAs interact with DNA, RNA, and protein and are involved in cancer phenotypes [13]. The lncRNAs MALAT1, HULC, HEIH, and HOTAIR are the most studied lncRNAs in HCC; HULC is a potential prognostic biomarker, and HEIH is closely related to HCC recurrence [14]. Other lncRNAs have been demonstrated to regulate ferroptosis during tumor development. Qi et al. [15] showed that, in HCC, erastin upregulated lncRNA GABPB1-AS1, which downregulated the translation of the GABPB1 transcription factor, thereby inhibiting the expression of the gene that encodes peroxidase, leading to cell death. Cases of predicting protein structures or testing genes based on bioinformatic methods have been reported and proved [16–18]. Increasing researches focused on predicting cancer prognosis and therapy response by transcriptomic analysis [19–21]. The prognostic potential of lncRNAs have been recently proved via bioinformatic analysis and presented impressive results [22, 23]. However, there are still limited studies based on ferroptosis-related lncRNAs and liver cancer. We developed a ferroptosis-related lncRNA (FRlncRNA) signature based on the expression of lncRNAs and explored the role of FRlncRNAs in tumor prognosis, immune infiltration, and genome instability. A risk model was constructed and validated in two separated cohorts, which showed the reliability of the FRlncRNA signature and indicated its potential as a prognostic biomarker in HCC treatment.

## 2. Materials and Methods

### 2.1. Data Collection

Transcriptome profiles converted into fragments per kilobase million (FPKM) together with clinical data of 364 patients with HCC, and the somatic mutation data of 350 patients with HCC were downloaded from the National Cancer Institute GDC Data Portal (Project ID: TCGA-LIHC [The Cancer Genome Atlas-Liver HCC dataset]) (https://portal.gdc.cancer.gov/repository). Complete lncRNA expression levels and survival data (follow-up with 0 day was excluded) were available for the 364 patients included in the study. The GTF main annotation file was downloaded from GENCODE (https://www.gencodegenes.org) and used to differentiate between mRNAs and lncRNAs. A total of 270 ferroptosis-related genes (FRGenes) were retrieved from FerrDb (http://www.zhounan.org/ferrdb/) [24] and previous publications [25, 26]. Full details of the FRGenes are provided in Table S1. FRlncRNAs were extracted by co-expression analysis based on the expression levels of lncRNAs and FRGenes. LncRNAs with correlation coefficients >0.4 and *P* < 0.001 were included in further analysis.

### 2.2. Construction of Prognostic Ferroptosis-Related lncRNA Signature

Patients were divided randomly into a training cohort and a test cohort. A risk model was established based on the training cohort and validated in test cohort. Cox regression, also known as proportional hazards model, is a survival analysis model to analyze the relationship between various features and survival time [27]. Least Absolute Shrinkage and Selection Operator (LASSO) is a commonly used regularization in many regression analysis methods for variable selection and shrinkage in Cox's proportional hazards model [28]. Lasso penalized Cox regression analysis have been widely used to construct gene expression based signatures in various cancers [29–31]. In our study, the FRlncRNAs were used as input of Lasso Cox regression to construct a FRlncRNA signature. Firstly, we performed univariate Cox analysis using the “survival” R package to identify FRlncRNAs that may have prognostic value. Then, LASSO method was carried out by “glmnet” R package to avoid overfitting. The correlation between the FRlncRNAs and FRGenes was visualized using Cytoscape [32]. The risk score is calculated as risk score = e^sum (expression × corresponding coefficient for each gene)^, where expression was the expression levels of the lncRNAs from the TCGA-LIHC dataset and the corresponding coefficient was calculated by LASSO penalized Cox regression analysis. Patients were divided into high- and low-risk groups based on the medium value of the risk score. Principal component analysis (PCA) and t-distributed stochastic neighbor embedding (t-SNE) analysis were then carried out using the “ggplot2” and “Rtsne” R packages, respectively. The risk score and survival status of each patient in the high- and low-risk groups were shown individually using the “pheatmap” R package.

### 2.3. Evaluation of Risk Model and Construction of a Predictive Nomogram

We performed a Kaplan-Meier analysis of the high- and low-risk groups and time-dependent receiver operating characteristic (ROC) curve to evaluate the predictive ability of the risk model and visualized the results using the “survival,” “survminer,” and “timeROC” R packages. The FRlncRNA signature was estimated and compared with the clinicopathological features by ROC, C index, and decision curve analysis using the “rmda,” “survival,” and “survivalROC” R packages. The clinical characteristics together with the risk score were used to set up a predictive nomogram using the “rms,” “foreign,” and “survival” R packages. The calibration curve to evaluate the nomogram was carried out by “rms,” “foreign,” and “survival” R packages.

### 2.4. Functional Enrichment Analysis

Differentially expressed FRGenes (DEGs) between the high- and low-risk groups were identified using the “limma” R package with false discovery rate (FDR) < 0.05 and the log of the fold change (∣logFC∣) > 1. All the identified DEGs were functionally annotated with gene ontology (GO) terms and Kyoto Encyclopedia of Genes and Genomes (KEGG) pathways using the “clusterProfiler,” “http://org.Hs.eg.db,” “enrichplot,” and “ggplot2” R packages.

### 2.5. Estimation of Immune Status and Genome Instability

CIBERSORT and single-sample gene set enrichment analysis (ssGSEA) were used to analyze the relationship between risk score and immune status [33]. We retrieved totally 12584 mutated genes of 350 patients from TCGA-LIHC database. Patients were divided into high- and low-risk groups according to the FRlncRNA signature. The mutation frequency in each group and different mutated genes between two groups were analyzed and visualized by “maftools” R package. Fisher's exact test was used to identify the significance of differently mutated genes.

### 2.6. Statistical Analysis

Co-expression analysis to identify FRlncRNAs and the relationships between variables was performed using the Pearson test. *P* values were adjusted by the Benjamini-Hochberg method to get the FDR. The chi-square test was used to compare differences in characteristics and risk score between the high- and low-risk groups. The clinical characteristics were analyzed by univariate and multivariate Cox analyses to identify independent prognostic factors. R software (version 4.0.3) and SPSS (version 18.0) were used for the data analysis. *P* <0.05 was considered statistically significant, except for those specified mentioned.

## 3. Results

**Figure 1:**
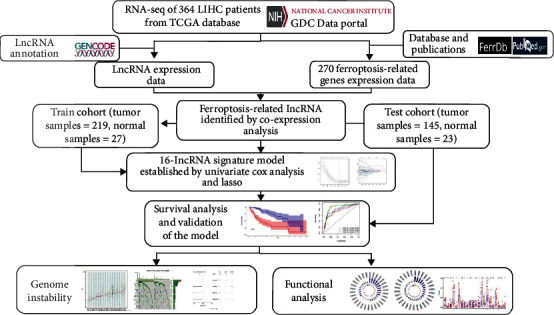
Flowchart of the data collection and analysis processes used in this study. LIHC: liver hepatocellular carcinoma, TCGA: The Cancer Genome Atlas.

A total of 364 patients for whom full expression and clinical data were available were enrolled in this study. A flowchart of the study design is shown in Figure 1.

### 3.1. Construction of a Ferroptosis-Related lncRNA Prognostic Signature

**Figure 2:**
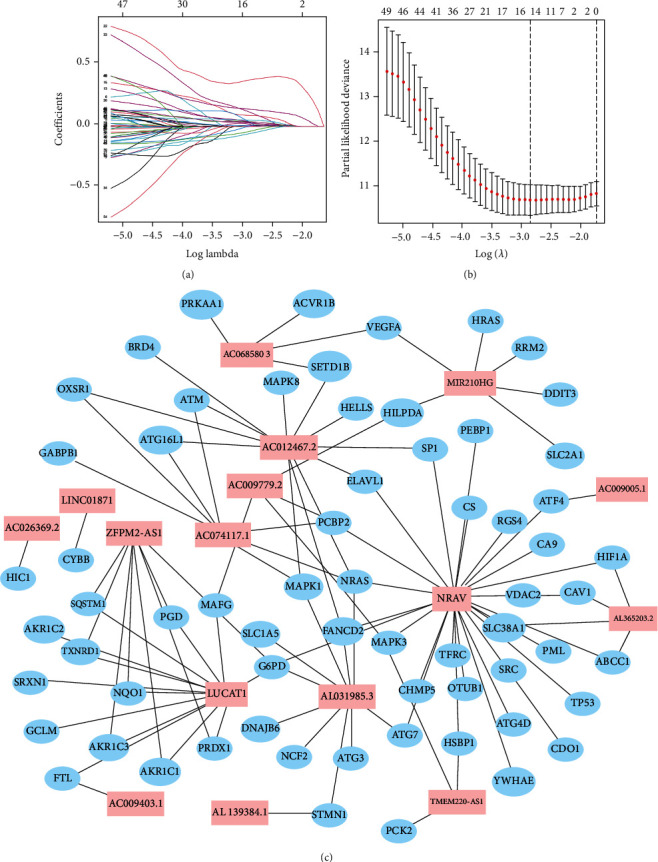
Establishment of the risk model. (a) LASSO coefficient profiles of 16 prognosis-related lncRNAs. (b) LASSO regression with tenfold cross-validation. (c) Interactions between the 16 differentially expressed ferroptosis-related lncRNAs (red) between tumor and adjacent normal tissue and ferroptosis-related genes (blue).

**Figure 3:**
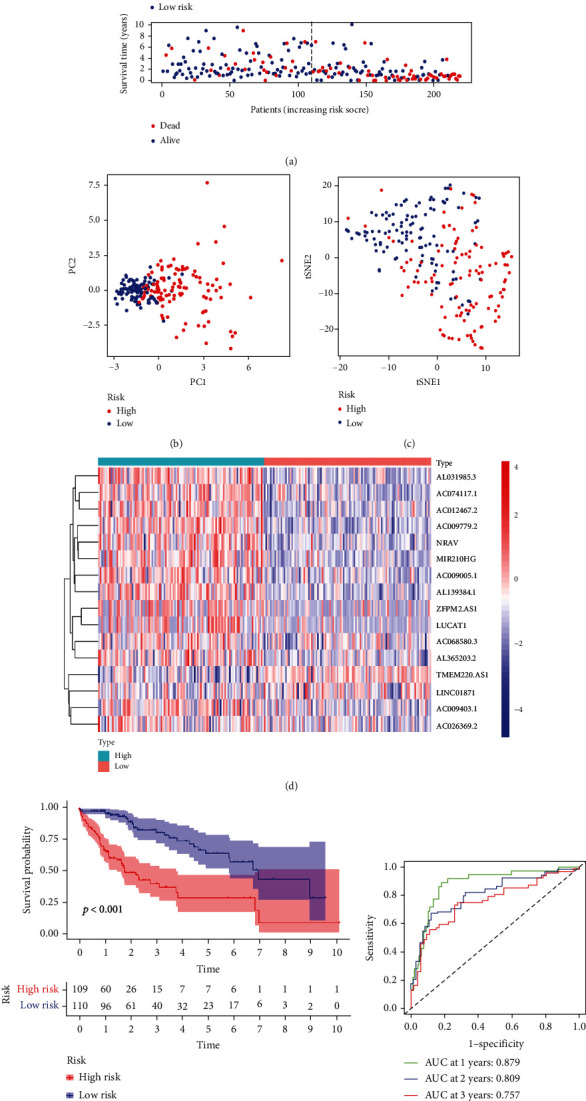
Prognostic analysis of the ferroptosis-related lncRNA signature model in the training cohort. (a) Risk survival status plot. (b) Principal component analysis plot. (c) t-distributed stochastic neighbor embedding (tSNE) analysis plot. (d) Heatmap of the expression of the lncRNAs. (e) Kaplan-Meier curves for overall survival. (f) AUC of time-dependent ROC curves. PC: principal component, ROC: receiver operating characteristic, AUC: area under the ROC curve.

The co-expression analysis identified 626 lncRNAs as FRlncRNAs (Table S2). Patients were divided randomly into a training cohort (*n* = 219) and a test cohort (*n* = 145). The clinical characteristics of the patients in the two cohorts are shown in Table 1. The univariate Cox analysis identified 54 lncRNAs that were significantly associated with HCC prognosis (Table S3). LASSO penalized Cox regression analysis was performed to construct the risk model (Figures 2(a) and 2(b)). Finally, 16 FRlncRNAs that were differentially expressed between tumor and adjacent normal tissue (AC009779.2, ZFPM2-AS1, AC009005.1, AC074117.1, AC012467.2, AL031985.3, AC009403.1, LUCAT1, AC026369.2, AC068580.3, LINC01871, AL139384.1, TMEM220-AS1, NRAV, AL365203.2, and MIR210HG) were identified and used to establish the prognostic FRlncRNA signature. The risk score was calculated as described in the method section. The corresponding coefficients of the FRlncRNAs and risk score for each patient are listed in Table S4. The interactions between the 16 FRlncRNA and FRGenes are shown in Figure 2(c). The training cohort was divided into high- and low-risk groups based on the medium value of the risk score, and the patients in the high-risk group were found to have higher mortality than the patients in the low-risk group (Figure 3(a)). The PCA and t-SNE analysis showed the separation of the two groups after features dimensionality reduction (Figures 3(b) and 3(c)). The differential expression levels of the FRlncRNAs between high- and low-risk groups show that most of them were positively correlated with the risk model (Figure 3(d)). The Kaplan-Meier analysis indicated that patients in the high-risk group had poorer overall survival (OS) (Figure 3(e); *P* < 0.001). The predictive performance of the risk model was evaluated by drawing a time-dependent ROC, where the area under the ROC curve (AUC) at 1, 2, and 3 years was 0.879, 0.809, and 0.757, respectively, in the training cohort (Figure 3(f)).

### 3.2. Validation of the FRlncRNA Signature in the Test Cohort

**Figure 4:**
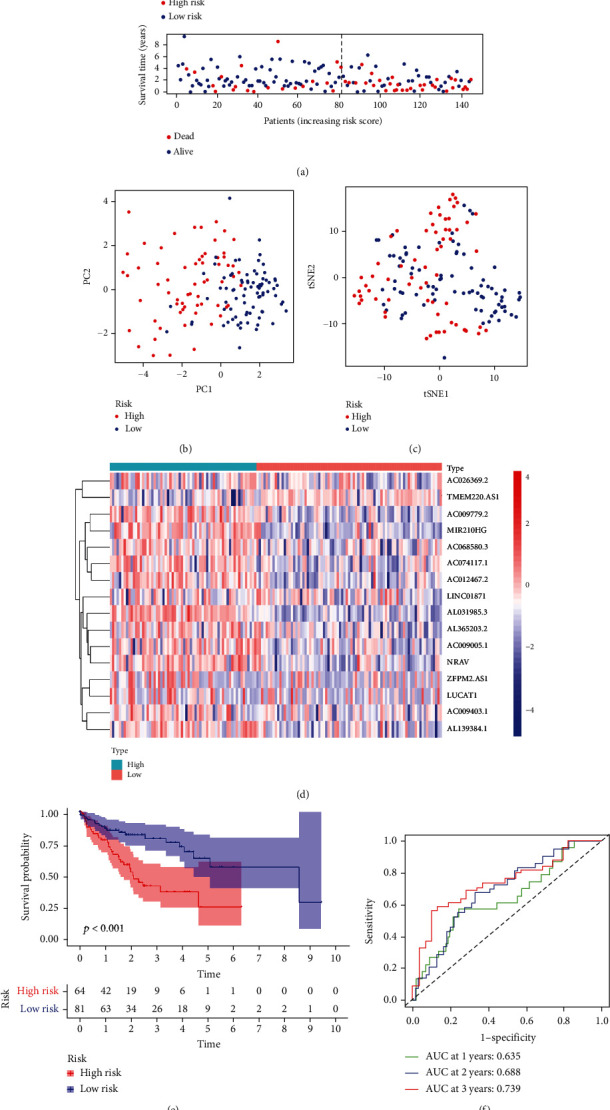
Prognostic analysis of the ferroptosis-related signature model in the test cohort. (a) Risk survival status plot. (b) Principal component analysis plot. (c) t-distributed stochastic neighbor embedding analysis plot. (d) Heatmap of the expression of the lncRNAs. (e) Kaplan-Meier curves for overall survival. (f) AUC of time-dependent ROC curves, PC: principal component, ROC: receiver operating characteristic, AUC: area under the ROC curve.

To test the robustness of the FRlncRNA signature model, we divided the test cohort into high- and low-risk groups using the same method that we used to divide the training cohort. The distribution of OS status and risk scores is shown in Figure 4(a). The PCA and t-SNE analysis confirmed that the patients in high- and low-risk groups were distributed in discrete directions (Figures 4(b) and 4(c)). The heatmap showed the detailed expression of the FRlncRNAs in the test cohort (Figure 4(d)). The Kaplan-Meier survival curve analysis showed that the patients in the high-risk group had significantly lower survival rates than the patients in the low-risk group (Figure 4(e)). AUC scores of 0.635, 0.688, and 0.739 at 1, 2, and 3 years, respectively, were obtained in the test cohort (Figure 4(f)).

### 3.3. Survival Analysis and Evaluation of the Ferroptosis-Related lncRNA Prognostic Signature

**Figure 5:**
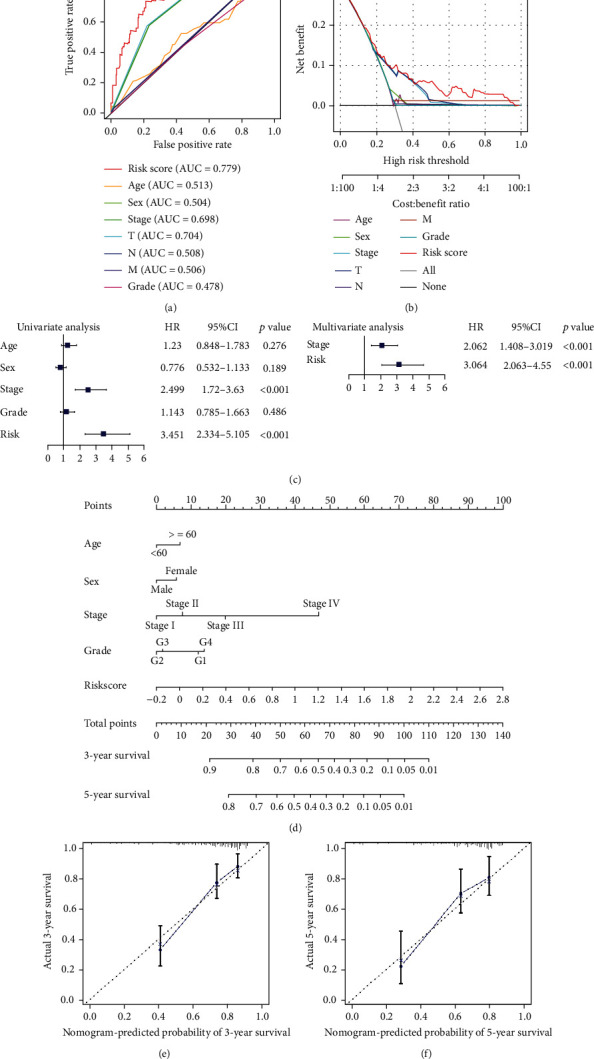
Assessment of risk factors and nomogram. (a) AUC values of various risk factors. (b) Decision curve analysis of the risk factors. (c) Univariate and multivariate Cox analysis for risk factors. (d) Nomogram for risk score and other risk factors. AUC: area under the ROC curve, T: tumor, N: nodes, M: metastases. (e–f) Calibration curves of nomogram for 3- and 5-year survival.

Patients in the high-risk group were correlated with higher tumor stage and tumor grade than patients in the low-risk group (Table 2). Then, patients from high- and low risk groups were combined to further evaluate the risk model. Compared with the prognosis predictions using age (AUC = 0.513 and C index = 0.512), sex (AUC = 0.504 and C index = 0.510), cancer stage (AUC = 0.698 and C index = 0.643), cancer grade (AUC = 0.478 and C index = 0.506), and the TNM Classification of Malignant Tumors staging system (AUC = 0.704, 0.508, and 0.506; C index = 0.647, 0.504, and 0.511), the prognosis prediction of the FRlncRNA signature was excellent (AUC = 0.779 and C index = 0.733) (Figure 5(a), Table 3). The decision curve analysis confirmed these results (Figure 5(b)). To determine the independent prognostic value of the risk model, the clinical characteristics of the patients in the high- and low-risk cohorts were examined by univariate and multivariate Cox analyses. The results identified the risk model (hazard ratio = 3.064 and 95%CI = 2.063–4.554) and tumor stage (hazard ratio = 2.062 and 95%CI = 1.408–3.019) as independent predictors of OS in patients with HCC (Figure 5(c)). The FRlncRNA signature and available clinicopathological features were used to establish a nomogram for OS prediction (Figure 5(d)) for application in clinical management of HCC. The calibration curves of 3- and 5-year OS showed good agreement with the survival prediction and the actual outcomes (Figures 5(e)–5(f)).

To further evaluate the FRlncRNA signature, we compared our risk model with reported risk models based on immune- or ferroptosis-related lncRNAs to predict prognosis of HCC. The formulae to calculate risk scores were retrieved from the publications and recalculated in our dataset [34–36]. Our signature showed better prediction ability (AUC = 0.779) compared to other three models (AUC = 0.729, 0.750 and 0.764) (Figure S1). By integrating risk score with clinicopathological features, our model also exhibited better prediction ability than using traditional pathological features (Table 3) [37, 38].

### 3.4. Estimation of Genome Instability with the Risk Model

**Figure 6:**
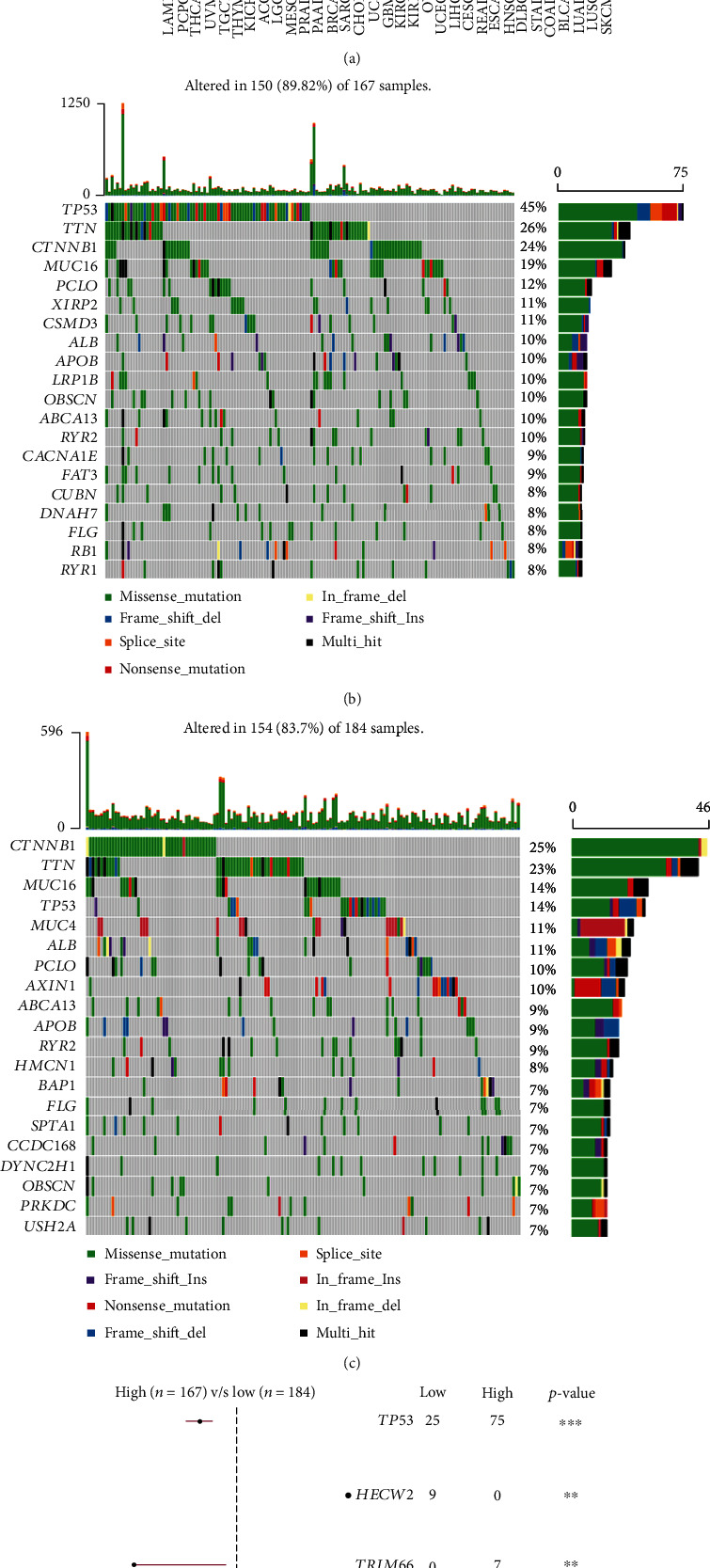
Analysis of genome instability. (a) Comparison of tumor mutation burden (TMB) of LIHC (black dots) with that of other cancers (grey dots) in The Cancer Genome Atlas. (b) Top 20 mutated ferroptosis-related genes in the high-risk group. (c) Top 20 ferroptosis-related mutated genes in the low-risk group. (d) Top 5 differentially mutated ferroptosis-related genes between the high- and low-risk groups. ^∗∗^*P* < 0.01 and ^∗∗∗^*P* < 0.001.

Considering the crucial role of somatic mutation in tumor initiation, development, and drug resistance, we also explored genome instability using the risk model. First, we compared the tumor mutation burden of liver HCC in the TCGA-LIHC dataset with that of 32 other cancers in TCGA (Figure 6(a)). The top 20 FRGenes with high mutation frequencies in the high- and low-risk groups are shown in Figure 6(b) and 6(c). Further analysis detected a total of 61 FRGenes with different mutation frequencies between the two groups (Table S5). Among them, the mutations in *TP53*, *HECW2*, *TRIM66*, *MCTP2*, and *KIAA1551* were significantly different (Figure 6(d); *P* < 0.01). The *HECW2* mutation frequency was higher in the low-risk group, and the *TP53* mutation frequency was the highest in high-risk group, and the three other FRGenes had higher mutation rates in the high-risk group than they had in the low-risk group.

### 3.5. Functional Analysis of the DEGs

**Figure 7:**
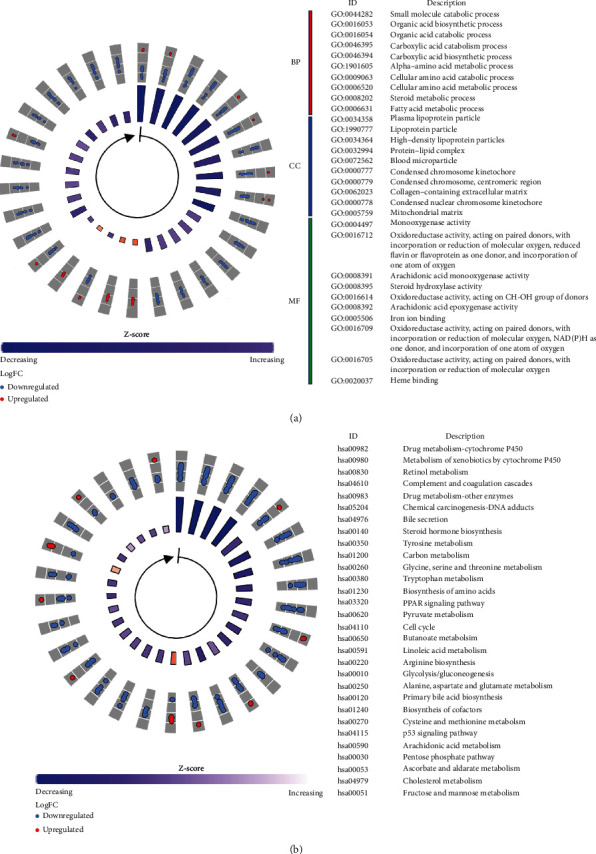
Representative results of the functional enrichment analysis of the differentially expressed ferroptosis-related genes. (a) Most significant enriched gene ontology terms. (b) Most significant enriched KEGG pathways. GO: gene ontology, BP: biological process, CC: cellular component, MF: molecule function, KEGG: Kyoto Encyclopedia of Genes and Genomes.

DEGs were identified between the high- and low-risk groups. GO and KEGG functional analysis was performed to explore their biological characteristics (Tables S6 and S7). Under the GO biological process category, the highly enriched terms included small molecule catabolic process, organic acid biosynthetic process, organic acid catabolic process, carboxylic acid catabolic process, and carboxylic acid biosynthetic process (Figure 7(a)). Under the GO cellular component category, the highly enriched terms included plasma lipoprotein particle, lipoprotein particle, high-density lipoprotein particle, protein-lipid complex, and blood microparticle (Figure 7(a)). Under the GO molecule function category, the highly enriched terms included monooxygenase activity, oxidoreductase activity acting on paired donors, atom of oxygen, steroid hydroxylase activity, and oxidoreductase activity acting on CH-OH group of donors (Figure 7(a)). The highly enriched pathways in the KEGG analysis included the drug metabolism-cytochrome P450, metabolism of xenobiotics by cytochrome P450, retinol metabolism, complement and coagulation cascades, and drug metabolism—other enzyme pathways (Figure 7(b)).

### 3.6. Estimation of Immune Status with the Risk Model

**Figure 8:**
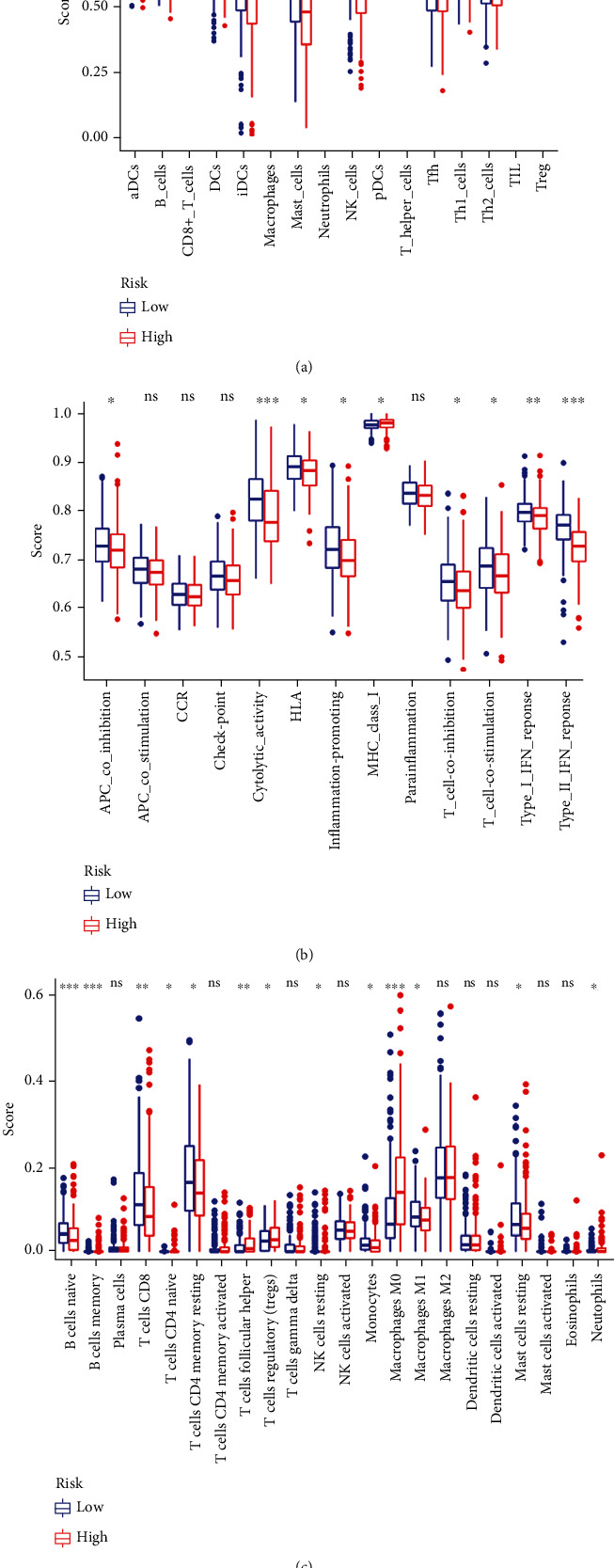
Estimation of the immune responses with the risk model. (a, b) Risk scores of immune cells (a) and immune-related functions (b) based on the single-sample gene set enrichment analysis. (c) Risk scores of immune cells based on the CIBERSORT analysis. ^∗^*P* < 0.05, ^∗∗^*P* < 0.01, and ^∗∗∗^*P* < 0.001.

Immunotherapy, which aims to mobilize the immune system to fight cancer, has drawn a lot of attention. To assess the association between the risk model and features of the immune cells, we performed ssGSEA (Figures 8(a) and 8(b)) and CIBERSORT analysis (Figure 8(c)). By combining the results, we found that B cells, CD8+ T cells, NK cells, and macrophages M1 were enriched in the tumor microenvironment of patients in the low-risk cohort. The ssGSEA also showed better immune function in patients in the low-risk group (Figure 8(b)).

On the basis of these results, we speculated that the 16 FRlncRNAs in the FRlncRNA signature may be associated with immune cells. We analyzed the relationship between FRlncRNAs with NK cells, B cells, CD8+ T cells, and M1 cells. Among the 16 FRlncRNAs, LINC01871 and AC026369.2 had the strongest correlation with the immune cells. The results of the correlation analysis are shown in Table S8.

## 4. Discussion

Inducing apoptosis to eradicate cancer cells has been the mainstay in clinical cancer treatment for a long time. However, resistance mechanisms have limited its implementation [39]. Ferroptosis, as an alternative process for cell death, has become a research hotspot to circumvent the resistance of cancer cells to apoptosis induction. Ferroptosis inducing drugs are associated with OS of cancer patients, and therefore targeting ferroptosis directly or triggering ferroptosis in combination with other therapies, such as immunotherapy or radiotherapy, may help to broaden the therapeutic armamentarium for anti-cancer strategies [12].

In this study, we set up an FRlncRNA signature that combined 16 differentially expressed FRlncRNAs to predict the prognosis of HCC. Briefly, we identified 626 FRlncRNAs and analyzed the relationship between the FRlncRNAs and OS. Sixteen of the FRlncRNAs were selected to establish the risk model in the training cohort, and the model was validated in the test cohort. The risk model was assessed by ROC and decision curve analysis. Although most of the FRlncRNAs in the signature have not previously been reported, some are associated with cancer development. A recent study showed that ZFPM2-AS1, which was upregulated in HCC, promoted HCC cell proliferation, invasion, and metastasis through the ZFPM2-AS1–miR-139–*GDF10* axis [40]. LUCAT1 was found to participate in the development and drug resistance of various tumors [41]. The LUCAT1–miR-5582-3p–*TCF7L2* axis increased the stem-like properties of breast cancer cells and stemness of breast cancer stem cells via the Wnt/*β*-catenin pathway, and LUCAT1 expression was related to tumor size, lymph node metastasis, TNM staging, and shorter OS in breast cancer [42]. Similarly, data analysis showed that high expression of LUCAT1 was associated with poor OS and relapse-free survival in HCC [43]. LUCAT1 was also confirmed to promote proliferation and metastasis in HCC *in vitro* and *in vivo* and to facilitate tumorigenesis by inhibiting ANXA2 phosphorylation [44]. MIR210HG, which acts as an oncogene in multiple tumors, was shown to promote cervical cancer progression through the MIR210HG–miR-503-5p–*TRAF4* axis, participate in methylation of the *CACNA2D2* promoter region to accelerate tumorigenesis of non-small cell lung cancer, and increase glycolysis-dependent oncogenic activity by potentiating the metabolic transcription factor hypoxia-inducible factor 1*α* in triple-negative breast cancer [45–47]. However, the role of MIR210HG in HCC is still unclear, calling for further exploration. HCC was found to have high tumor mutation burden, and therefore we explored its genome instability. We compared the somatic mutation data of patients in the high- and low-risk groups and found that the mutation frequency of *TP53* was significantly increased in the high-risk group compared with the frequency in the low-risk group (45% versus 25%). *TP53*, which is a known tumor suppressor gene, had a lower mutation frequency in the low-risk group, which may explain the better prognosis for patients in the low-risk group. *TRIM66* expression has been shown to promote malignant progression in several types of cancer, including HCC [48, 49], and MCTP2 inhibited cisplatin resistance in gastric cancer [50]. The results of the KEGG analysis indicated that the drug metabolism function differed in the high- and low-risk groups, implying that patients in the high-risk group may potentially be less sensitive to chemotherapy. KIAA1551 was also annotated as a tumor suppressor. On the basis of these findings, we believe that our risk model can predict not only OS but also resistance of chemotherapy [51].

The GO and KEGG analysis indicated that, as expected, the DEGs were enriched in fatty acid, lipid and redox reaction, which are associated with ferroptosis [52]. Among the many other metabolic processes, drug metabolism ranked first in the KEGG analysis. Liver is the primary organ of biotransformation, which involves various biotransformation enzymes. Members of the cytochrome P450 family (CYP450) are the main xenobiotic-metabolizing enzymes that play vital roles in drug metabolism. All the members of the CYP450 superfamily were shown to be significantly downregulated in HCC tissues compared with their expression in normal tissues [53], and two isoforms (CYP2C9 and CYP2E1) were found in lower abundance in high-grade HCC tumors, implying that substrates such as antitumor drugs may be eliminated more slowly and achieve higher concentrations [53]. Sorafenib has been approved as first-line treatment for HCC. Sorafenib metabolism was shown to be significantly decreased in tumor hepatic microsomes together with the downregulation of *CYP3A4* and *UGT1A9* expression [54]. However, although the low metabolism of sorafenib increased its bioavailability, it also caused toxic effects such as hand and/or foot skin reactions under normal drug doses [54]. Therefore, the activity of the CYP450 family should be considered when prescribing these drugs, and the FRlncRNA signature may guide clinical treatments.

Cancers not only consist of malignant cells but also recruit other cells such as stromal cells, extracellular matrix, and immune cells, which together make up the tumor microenvironment (TME) [55]. Immune cells, including innate and adaptive immune cells, have recently become the focus of much attention in the context of cancer, and T cells have been deemed to play a vital role in the anti-cancer immune response. For a long time, CD8+ T cells have been considered to mediate antitumor responses in the tumor immune microenvironment, indicating that patients in the low-risk group tended to have a better antitumor response [56]. We also found more T helper cells and NK cells in the low-risk group. T helper cells and NK cells are vital parts of the immune system and components of the TME and their roles have been elucidated in many studies [57, 58]. Interestingly, we also found more B cell infiltration in the low-risk group, and recent studies have shown that B cells also participate in the immune response [59–61]. Fridman et al. [60] considered that enrichment of B cells and tertiary lymphoid structures was the strongest prognostic factor of prolonged survival and was positively correlated with the response to PD-1 blockade in soft-tissue sarcomas. Together, these studies showed that B cells were not just bystanders in antitumor immunotherapy; indeed, the presence of B cells has provided a new target for immunotherapy and could be a strong weapon against tumors. Nevertheless, the detailed mechanisms of immune cells in the TME are still unclear, although some studies have shown that lncRNAs participate in various processes of the immune response in the TME [62]. For example, overexpression of lncRNA HOTAIR in HCC cell lines promoted CCL2 secretion, which is necessary for tumor-associated macrophages and recruitment of myeloid-derived suppressor cells [63]. Given the prominence of tumor-associated macrophages, the lncRNAs GNAS-AS1, Xist, and MMA2P were also shown to regulate M2 polarization, thereby contributing to tumorigenesis [64–67]. Although T cell infiltration is a major property of the TME, lncRNAs have been demonstrated to be involved in the regulation of cytotoxic T lymphocytes. Upregulation of the lncRNAs NEAT1, lnc00473, and SNHG14 was associated with immune evasion by inhibiting T cell infiltration and suppressing the activation of cytotoxic T lymphocytes [68–70]. In this study, we also showed the association between FRlncRNAs and immune cells. Indeed, all the FRlncRNAs were involved in the association with immune cells to some extent, and LINC01871 and AC026369.2 showed the most significant correlations, especially with CD8+ T cells and B cells. The antitumor effect of CD8+ T cells is clear, and their reported presence suggests that memory B cells and germinal center B cells might be involved in the ongoing formation of tertiary lymphoid structures. Our results provide evidence that the prognostic FRlncRNA signature may have the potential to predict efficacy of immunotherapy and are worth further study to improve the TME.

Overall, as ferroptosis has become a new therapeutic target to attack tumors, numerous studies have been conducted or are underway. In this study, we explored the association between FRlncRNAs and OS of patients with HCC. An FRlncRNA signature that combined 16 FRlncRNAs was established to predict the prognosis of HCC. However, the risk model needs to be validated in more cohorts. The deeper mechanisms among FRlncRNAs, immunity, and genome instability are still unclear. The potential capability of the FRlncRNA signature to instruct clinical treatment also deserves further study. In addition, it is worth promoting application of analyzing a few gene data to reduce the cost of sequencing [71]. It would be much better if the outcome, mutated and immune related events can be predicted only by measuring these FRlncRNAs expression. Earlier studies have proposed database to predict prognosis, such as a Human papillomavirus (HPV) genotype prediction tool, which can predict HPV carcinogenic or non-carcinogenic risk genotypes [72]. In the future, we would further design similar database to better show our signature.

## 5. Conclusion

The risk model of 16 ferroptosis-related lncRNAs is closely related with overall survival, tumor immune environment, and genome instability in hepatocellular carcinoma.

## Figures and Tables

**Table 1:** Clinical characteristics of the patients obtained from TCGA-LIHC dataset.

No. of patients	Train cohort	Test cohort
	219	145
Age (median [IQR])	62.00 [51.00, 70.00]	61.00 [52.00, 68.00]
Sex (%)		
Female	73 (33.3)	46 (31.7)
Male	146 (66.7)	99 (68.3)
Stage (%)		
Stage I	106 (48.4)	63 (43.4)
Stage II	52 (23.7)	32 (22.1)
Stage III	46 (21.0)	37 (25.5)
Stage IV	—	4 (2.8)
Unknown	15 (6.8)	9 (6.2)
*T* (%)		
T1	114 (52.1)	65 (44.8)
T2	54 (24.7)	37 (25.5)
T3	42 (19.2)	36 (24.8)
T4	6 (2.7)	7 (4.8)
Unknown	3 (1.4)	—
*N* (%)		
N0	143 (65.3)	104 (71.7)
N1	2 (0.9)	2 (1.4)
Unknown	74 (33.8)	39 (26.9)
*M* (%)		
M0	155 (70.8)	107 (73.8)
M1	—	3 (2.1)
Unknown	64 (29.2)	35 (24.1)
Grade (%)		
G1	34 (15.5)	21 (14.5)
G2	110 (50.2)	65 (44.8)
G3	66 (30.1)	51 (35.2)
G4	6 (2.7)	6 (4.1)
Unknown	3 (1.4)	2 (1.4)
Vital status (%)		
Alive	140 (63.9)	94 (64.8)
Dead	79 (36.1)	51 (35.2)

TCGA: The Cancer Genome Atlas, LIHC: liver hepatocellular carcinoma, IQR: interquartile range.

**Table 2:** Baseline characteristics of the patients in the high- and low-risk groups.

Characteristics	High risk	Low risk	*p* value
Total			
364			
Age (%)			0.471
<60 (year)	75 (43.4)	90 (47.1)	
≥60 (year)	98 (56.6)	101 (52.9)	
Sex (%)			0.32
Female	61 (35.3)	58 (30.4)	
Male	112 (64.7)	133 (69.6)	
Stage (%)			0.02
I + II	108 (62.4)	145 (75.9)	
III + IV	51 (29.5)	36 (18.8)	
Unknown	14 (8.1)	10 (5.2)	
Tumor grade (%)			<0.001
G1 + G2	90 (52.0)	140 (73.3)	
G3 + G4	81 (46.8)	48 (25.1)	
Unknown	2 (1.2)	3 (1.6)	

**Table 3:** C indexes of nomograms and clinicopathological features.

	C index	95% CI
Age	0.512	0.438-0.586
Stage	0.643	0.580-0.706
Grade	0.506	0.435-0.577
Sex	0.510	0.453-0.567
*T*	0.647	0.584-0.710
*N*	0.504	0.490-0.518
*M*	0.511	0.495-0.527
Risk score	*0.733*	0.672-0.794
Nomogram	*0.755*	0.700-0.810
Reported nomogram 1 (primary cohort) [37]	0.661	0.633–0.688
Reported nomogram 1 (validation cohort) [37]	0.657	0.626-0.698
Reported nomogram 2 (primary cohort) [38]	0.667	0.653-0.681
Reported nomogram 2 (validation cohort) [38]	0.663	0.640-0.686

## Data Availability

Publicly available datasets were analyzed in this study. This data can be found here: TCGA database (https://portal.gdc.cancer.gov/repository), GENCODE (https://www.gencodegenes.org), and FerrDb (http://www.zhounan.org/ferrdb/).

## Supplementary Materials

Supplementary Materials Table S1. Details of the 270 ferroptosis-related genes retrieved from FerrDb. Table S2. The 626 lncRNAs identified as ferroptosis-related lncRNAs by co-expression analysis. Table S3. The 54 lncRNAs that were significantly associated with hepatocellular carcinoma prognosis by univariate Cox analysis. Table S4. Corresponding coefficients of ferroptosis-related lncRNAs and risk score. Table S5. The 61 ferroptosis-related genes with different mutation frequencies that were found by comparing the high- and low-risk groups. Table S6. Detailed results of the GO functional enrichment analysis. Table S7. Detailed results of KEGG pathway enrichment analysis. Table S8. Association between 16 ferroptosis-related lncRNAs and immune cells.
