# Supplementary figures and images for: A Ferroptosis-Related LncRNA Signature Associated with Prognosis, Tumor Immune Environment, and Genome Instability in Hepatocellular Carcinoma
Source: Comput Math Methods Med. 2022 Aug 18;2022:6284540. doi: 10.1155/2022/6284540 (PMC9410853; doi:10.1155/2022/6284540)

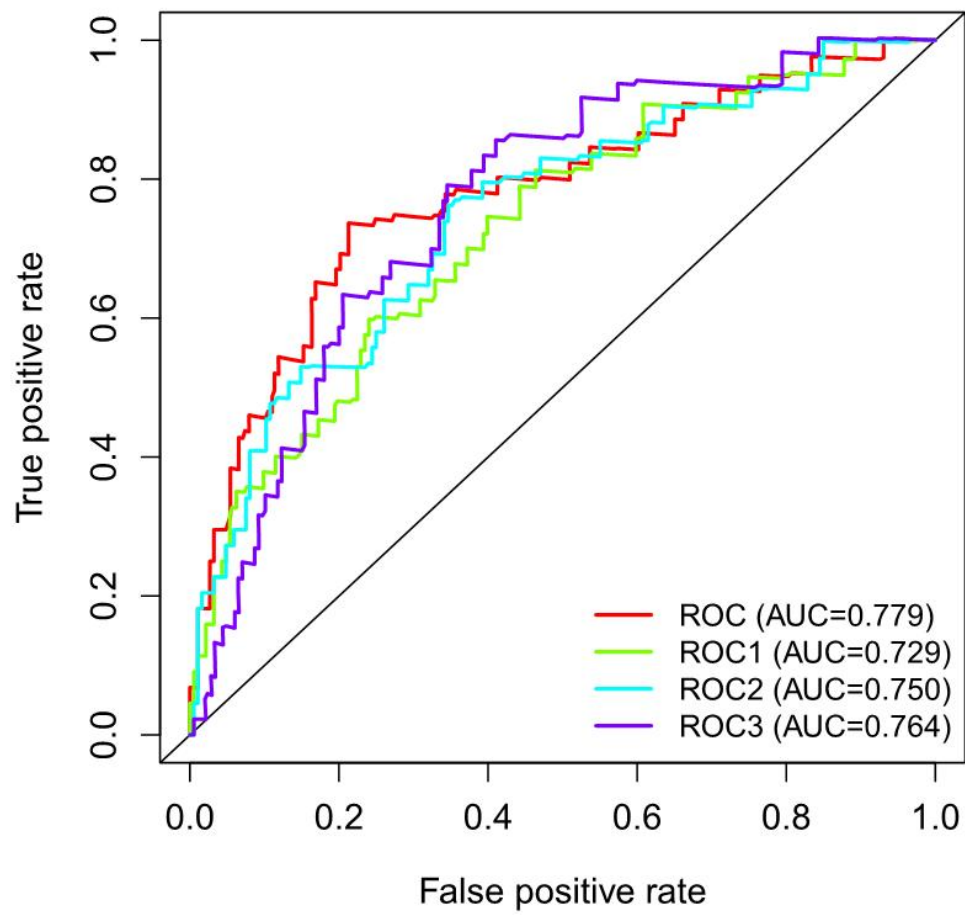

Fig.S1 The comparison of prediction ability between FRlncRNA signature and reported models [34-36].

Supplement: Supplementary Materials — Table S1. Details of the 270 ferroptosis-related genes retrieved from FerrDb. Table S2. The 626 lncRNAs identified as ferroptosis-related lncRNAs by co-expression analysis. Table S3. The 54 lncRNAs that were significantly associated with hepatocellular carcinoma prognosis by univariate Cox analysis. Table S4. Corresponding coefficients of ferroptosis-related lncRNAs and risk score. Table S5. The 61 ferroptosis-related genes with different mutation frequencies that were found by comparing the high- and low-risk groups. Table S6. Detailed results of the GO functional enrichment analysis. Table S7. Detailed results of KEGG pathway enrichment analysis. Table S8. Association between 16 ferroptosis-related lncRNAs and immune cells. [file 6284540.f1.zip › Supplementary figureS1.pdf]
